# Playful Communication and Care: Exploring Child-Centred Care of Young Children With Type 1 Diabetes Through the Framework of Zone of Proximal Development

**DOI:** 10.3389/fcdhc.2021.707553

**Published:** 2022-02-11

**Authors:** Patricia DeCosta, Dan Grabowski, Louise Norman Jespersen, Timothy C. Skinner

**Affiliations:** ^1^ Department of Psychology, University of Copenhagen, Copenhagen, Denmark; ^2^ Diabetes Management Research, Steno Diabetes Center Copenhagen, Copenhagen, Denmark; ^3^ Department of Rural Health, La Trobe University, Bendigo, VIC, Australia

**Keywords:** young children, type 1 diabetes, psychosocial care, zone of proximal development, play, child-centred care

## Abstract

**Introduction:**

Little is known about the psychosocial experiences and care needs of young children under the age of 7 years who have been diagnosed with type 1 diabetes. To address this knowledge gap, we examine children’s psychosocial care needs through the lens of child-centred care and the framework of Zone of Proximal Development.

**Objectives:**

To explore current care practices for young children with diabetes and identify aspects of child-centred care already successfully integrated into current practice.

**Method:**

Individual face-to-face, semi-structured interviews were conducted with 20 Healthcare Professionals, representing 11 of 17 paediatric diabetes clinics in Denmark.

**Results:**

Our data provided valuable insights into existing child-centred practices. Our analysis identified practices covering four main themes: 1. Accommodating immediate emotional needs, 2. Putting children before diabetes, 3. Encouraging meaningful participation, 4. Playful communication.

**Discussion:**

Healthcare Professionals provided child-centred care, largely through play-based approaches that make diabetes care meaningful and relevant. Such practices provide the scaffolding necessary to enable young children to gradually engage, comprehend and participate in their own care.

## Introduction

Type 1 diabetes is one of the most common chronic diseases diagnosed in childhood (1). Over the past 20 years, the number of children diagnosed with type 1 diabetes has been increasing (2), with the greatest increase observed in children under 5 years of age (3). However, little is known about the psychosocial needs and experiences of young children under the age of 7 years who have been diagnosed with type 1 diabetes (4). The lack of knowledge regarding this age group is reflected in *The Position Statement from the American Diabetes Association, on psychosocial care for people with diabetes*, which does not address children under the age of 7 years (5).

In the adult as well as child population, the importance of being sensitive to the broader psychosocial dimensions of living with a chronic illness has gained increased recognition (6). Accordingly, providing patient-centred care (PCC) has been a central objective in improving healthcare, as suggested by the US Institute of Medicine (7). While a significant amount of research on PCC currently exists, including systematic reviews, showing that PCC processes are positively correlated with patient satisfaction, well-being and clinical outcomes (6), the literature on child-centred care (CCC) in healthcare is still relatively new (8). Likewise, CCC is not mentioned in the Consensus Guidelines for *psychological care of children and adolescents with type 1 diabetes* (9). However, viewing the psychological care of children through a child-centred lens is needed to ensure that guidelines represent both a child perspective and the child’s insider perspective (10).

CCC encompass being responsive to children’s views and preferences, eliciting the child’s perspectives, tailoring care to the individual child’s needs, building relationships and providing children with the time and opportunity for participation (11). Thus, adapting a CCC approach in healthcare reflects a wider acknowledgement of children’s rights as well as their right to participate and be involved in their own healthcare (10). Research suggests that children favour CCC, although this need may not currently be accommodated (12). Consistently, a recent review, consolidating the views of at least 650 (mainly older) children with type 1 diabetes, found that children preferred individualised, collaborative, relationship-based diabetes care (13).

Accordingly, in the present study, we will address the gap in knowledge regarding young children’s (age <7 years) psychosocial care needs and do so through the lens of CCC. Specifically, we will accomplish this by exploring the knowledge and practical experience of healthcare professionals (HCPs) working with these young children.

Our objective is to explore current care practices for young children with type 1 diabetes and identify aspects of CCC already successfully integrated into current practice.

### Theoretical Framework

To ensure that our objectives are explored in an appropriate developmental context, we use Vygotsky’s theory of Zone of Proximal Development (ZPD) (14) and the concept of scaffolding (15) to frame our analysis. The ZPD can be described as ‘the distance between the actual developmental level as determined by independent problem solving and the level of potential development as determined through problem solving under adult guidance or in collaboration with more capable peers’ (14). Scaffolding refers to the activities and tools provided by an adult or capable peer that serve to support the child as he or she is led through the ZPD (15). The theory of ZPD acknowledges that development is intrinsically linked to learning, as opposed to the notion that learning can only take place when a certain developmental stage has been reached. From the ZPD perspective, even young children have the ability to understand, contribute to and participate in their own care, provided that interactions take place within the ZPD and are supported by the use of age-appropriate tools and activities.

In the context of young children, play is a fundamental part of the ZPD. Young children communicate, express emotion and comprehend information through play: ‘Play creates a zone of proximal development of the child. In play, the child always behaves beyond his average age, above his daily behaviour; in play, it is as if he is a head taller than himself. As in the focus of a magnifying glass, play contains all developmental tendencies in a condensed form and is itself a major source of development’ (14). Play is widely used in therapeutic settings to strengthen the connection and positive interaction between young children and their caregiver as well as to examine young children’s attachment and relationships (16, 17). Accordingly, we use the theory of ZPD, the concept of scaffolding and play as a framework both to identify CCC practices and to understand the mechanisms that make these practices appropriate from a developmental perspective.

## Method

HCPs from all paediatric diabetes clinics in Denmark (n=17) were invited to participate in this qualitative study. A total number of 20 HCPs, representing 11 clinics, accepted the invitation. One clinic declined, while two clinics did not respond. Another three clinics initially accepted the invitation. However, they did not respond to further attempts to arrange the interview. Individual face-to-face interviews were conducted with 11 paediatric nurses, four paediatricians, three psychologists and two dietitians. The HCPs experience in paediatrics ranged from 10 to 40 years (median 19 years). All interviews took place at the home clinic of the respective HCP and lasted between 23-50 minutes. We used an open-ended, semi-structured interview guide, created with the objective to study current care practices, explore knowledge and experiences of HCPs and gain insights into the needs and experiences of young children with type 1 diabetes. The guide included 18 open-ended questions concerning young children’s psychosocial needs, experiences, challenges and strengths, as well as current care practices. E.g., how do you involve young children and elicit their perspectives at diagnosis and in subsequent consultations? Have this practice changed over time?

Prior to data collection, the first author (PD) passively observed a number of paediatric diabetes consultations at a clinic in Denmark. While these observations were not used in the analysis, they provided a contextual background for the subsequent interviews. All interviews were conducted by PD. The data were transcribed verbatim by PD and a student assistant. The study took place between December 2018 and January 2020.

### Ethics

The study was approved by The Institutional Ethical Review Board, University of Copenhagen, Department of Psychology. Approval number: IP-IRB/14112018.

### Analysis

In accordance with Braun and Clarke (18), we used thematic analysis to identify and report patterns – or themes – in our interview data. A preliminary analysis of the data was conducted in Phase 1 by listening to the interviews and rereading the written transcripts. In Phase 2, we transferred the transcribed dataset into the qualitative data analysis program Nvivo for detailed coding. At this point, analysis was narrowed to identify CCC practices, as described by the HCPs. Specifically, we gathered all data in which HCPs described practices that not only fulfilled young children’s medical needs, but also supported their psychosocial needs. In Phase 3, we searched for potential themes by gathering and grouping codes, as well as pulling together all data relevant to each potential theme. In Phase 4, we refocused the analysis to the broader level of themes, and we constructed a thematic map. In Phase 5, themes were refined, defined and ultimately named. All authors (PD), (LNJ), (DG) and (TS) took part in the discussion throughout the analysis, and consensus regarding final themes was reached jointly.

## Results

Our data revealed significant diversity in practices, views and beliefs concerning young children’s need and ability to participate in their own care. Some HCPs found that cognitive and language abilities were a barrier to eliciting young children’s views in a meaningful way. Others found that participation was unnecessary, as the responsibility for diabetes care rested fully with the parents. A few HCPs simply did not put much emphasis on young children’s psychosocial needs or experiences, viewing diabetes care in this particular age group as principally biomedical.

‘And in that way, it may be easy, because when we typically have 2- to 3-year-olds, or a 4-year-old for that matter too, then of course it can be about us having to talk to them about that; “now you need this here” and “I know that it’s not that fun” and stuff like that. But if you put it a little more bluntly, then it is a little like ‘a body’ that has to line up for this. But it is the parents who really have to do it, and it is the parents you talk to, if that makes sense?’ – Paediatrician (10).

At the same time, others provided great insight into how to elicit young children’s perspectives and actively engage children in their own care. They described a coordinated strategy for being sensitive to and meeting young children’s needs, providing numerous examples of specific child-centred practices. The data describing such practices are presented here.

We identified four main themes in our analysis: accommodating immediate emotional needs, putting children before diabetes, encouraging meaningful participation and playful communication.

### Accommodating Immediate Emotional Needs

At diagnosis, the HCPs found that young children reacted to the immediate pain of blood tests and insulin injections, to the cues of the emotional state of their parents, as well as to being surrounded by strangers in an unfamiliar environment. The HCPs explained that the immediate need of young children, in addition to medical treatment, was to feel safe and become familiar with their new surroundings and the people treating them; a prerequisite for learning and development to occur. For the majority of children admitted to hospital, medical intervention could safely be delayed, or kept to a minimum, in order to accommodate the psychological needs of young children and their families.

‘Their greatest need, here and now, is that it should be safe and calm, and not dangerous. That is, when they are admitted. Let’s do a minimal intervention on them. Something good will come out of it. And something good will come out of that in the long run, as well’. – Paediatrician (19).

For young children in particular, pricks, jabs and injections were a major source of fear, anxiety and distress immediately following diagnosis. The HCPs with many years of experience could describe how an increased focus on children’s psychological experiences had changed practices over time. Now, whenever possible, they would increase the time between admittance and treatment in order to minimize the negative consequences associated with the experience of pain.

‘I think it’s a huge advantage that we don’t have to prick the kids as much anymore. And I also think that the general attitude has become, -in the past when a child with newly diagnosed diabetes came, we immediately swarmed them [… ] there was not much awareness of that, all these many jabs, it actually wasn’t good. Where now, we are much more hesitant, which we can be, because they often come to us well before they are in ketoacidosis. So we try to hold back a little bit with all these jabs. So there may well be a diabetes child who comes to us, where our assessment is, that the situation is such that we can easily put an Emla [anaesthetic patch] on and wait that hour to one and a half hours, until it has worked, and the lab technicians can come, before we even do anything at all. Um, I think that’s a major advantage and a major change’. –Nurse (1).

In response to children’s need to feel safe, the HCPs tried to create a framework of predictability and continuity, from the time of admission through transferring to outpatient clinic appointments. They would explain, or in the case of young children demonstrate, what was going to happen. They acknowledge that without a framework and age-appropriate explanations, young children had little understanding of the new situation in which they found themselves.

‘Yes, so I think they need some, things like a sense of trust, a feeling of safety, and a framework so that they know what’s going on in here, but also that they know what’s going to happen, and so on, and that things are explained … And time, right; that we’ve got the time for them too’. –Nurse (16).

In the acute phase of diagnosis, where learning and development was not the main objective, care practices operating within young children’s ZPD, still proved valuable. By demonstrating procedures, rather than relying on solely verbal communication, HCPs actively assisted children’s understanding with the intention of providing a predictable framework and a sense of safety.

One HCP described how their clinic had a coordinated strategy for diabetes education to be delivered consistently by the same nurse in the team, from admission onwards. This approach differed from that used at other clinics, where initial diabetes education was delivered by the HCPs on the ward. For caregivers, this approached insured that consistent information was given at all time. Importantly, for young children, it meant being able to build relationships with the people treating them.

‘We actually see them quite frequently during the hospitalisation, and it provides a really good foundation for the future collaboration we have, when we will see them a few times a year, when they come to our outpatient clinic. So that beginning, it’s a good investment’. –Nurse (16).

### Putting Children Before Diabetes

By anchoring social interaction and learning within the context of children’s everyday life, HCPs were more likely to engage young children in the ZPD and ensure child-centered practices. The HCPs emphasised the importance of seeing and acknowledging the whole child first, before putting any focus on diabetes. Accordingly, they viewed diabetes in the context of children’s everyday lives. The rational for and proposed benefits of this approach were manifold.

‘We would like them to understand that it is them as a person who is exciting and interesting and funny and that we are curious about. [… ] If they can come and tell me, like all other children, what they’re doing and what they did yesterday, or what they are going to do today, without talking about diabetes. Then it’s a sign that things are going well’. – Paediatrician (19).

In this way, the HCPs wished to convey the message that, even in the hospital or clinic setting, diabetes is only one part of the child’s life – and not the most important part. At the same time, the children’s inclination to talk about their everyday life revealed important information about their experience of living with diabetes, as well as giving an initial indication of adjustment.

‘It’s about the person being important. And that diabetes is, in quotation marks, “is just the circumstance”. It’s something you have to carry, and that we have to work with, but it’s not what’s ultimately important. It’s the person you are, and who you are today and tomorrow and in the future. And the wishes and dreams that you have, that’s what’s important. Because somehow, it’s not just about blood sugar, or it’s actually not about that it at all, it’s about quality of life, right’. – Paediatrician (19).

Putting diabetes in the context of young children’s everyday life and showing interest in their life outside the clinic mattered to even young children. The HCPs found that this practice laid the foundation for building relationships and trust. In this way, such practice was consciously integrated into the clinic appointment.

‘In fact, it’s important to us; if the child has told us something –it could be that he was going on an overnight fieldtrip with the kindergarten, some still do that, that we then note in their medical records. Because when they return next time, then we remember to ask about it; how did it go? So that they will go – Ok! They actually know me! It can be difficult for us to remember all the kids, so that’s why we make sure to write it down. So they get a sense that – they actually know me well. [… ] And I think I establish a good relationship with the families. Therefore, I think if there is something that they find really difficult, then they also come to me and tell me about it’. – Nurse (12).

Additionally, showing an interest in the whole child and what was important to that individual was viewed in relation to the long-term perspective of these children’s continued contact with the HCPs and the clinic. Although the interaction seldom centred on diabetes, but rather on what was meaningful to young children in their everyday life, it entailed important learning about how to actively engage and interact with HCPs in the consultation – an important skill for children with a chronic illness.

‘We also do it with the purpose to teach them to speak for themselves in the consultation. Because at some point when they get a little older, then they’re used to talking to us. And then it might also be easier to bring up other issues, things that they’re struggling with, in relation to their diabetes’. – Nurse (12). This practice illustrate how HCPs, who operate within young children’s immediate cognitive and psychological range, essentially utilize the ZPD. When HCPs encourage young children to express their understandings, likes and feelings concerning their everyday life, they guide and assist these children’s future ability to express their understandings, needs and feelings, in relation to diabetes management.

Integrating such practices as well as ensuring early and active engagement required active consideration and planning on the part of the diabetes team. The HCPs were aware that technology, and the instant data provided by it, could potentially direct attention away from the child sitting there in front of them.

‘So it takes a lot of time to get all these practical things done around the equipment itself. Then I also think that the more information you get from the pump and censor, and we read it, the more we become focused on this screen and the more focus moves away from the child. So we have to be really conscious to say: Well okay, it’s also important that we have time to look at the screen and adjust what needs to be adjusted, but it must not be at the expense of us forgetting the child and forgetting the family, and we forget to hear some of these soft values in the consultation as well. It’s all just very hard facts once you get things up on the screen with numbers’. – Dietitian (6).

The time invested in getting to know young children also gave the HCPs an insight into both the individual child’s emotional state and how such emotions were expressed. In turn, the HCPs could be responsive to the needs and preferences of even young children who lack the ability to fully express their experiences, verbally or nonverbally.

‘When I start to get to know the children, I know what works, that is, from child to child. So I know that, well there are some who may be quite hyper [behaviour] when they come. They don’t quite know, - I then know that we need some kind of diversion. There’s one boy for example, where I know that when he comes, he just needs to be allowed to say a lot of things, and sometimes there are some swear words and stuff like that. [… ] I think it’s his way of saying, that he thinks it’s a little annoying, that he has to come here’. – Nurse (13).

### Encouraging Meaningful Participation

There was consensus among the HCPs that young children quickly lost interest and got bored when adult attention was focused on the computer screen or exclusively ‘adult talk’. Evaluating data from the insulin pump or glucose meter is a fundamental part of the consultation. However, without modification, this activity is outside young children’s ZPD and does not offer them any real opportunity for participation. Children would show signs of boredom by becoming fidgety or restless. Because of this, some HCPs explained that they would make a clear distinction between the activities they sought to actively engage the children in and the activities clearly aimed at the caregivers.

‘So I often make a point of saying; you know what, now we’re actually done with this, so feel free to sit down with your iPad, or feel free to go out and play in the waiting room, or whatever you fancy. [… ] Because it often is the boring stuff, right? And it will be things they don’t understand. So I think it’s important that when we want their attention, it’s on activities they can participate in and find interesting. Also to keep up their spirits for the next time they have to come in for a consultation’. – Nurse (12).

In this way, the HCPs were responsive to young children’s preferences and only asked for their attention when they had a real opportunity to engage. They acknowledged that, if the activity did not actively include or engage the child, young children’s attention span was very limited. In addition, the HCPs emphasised that one prerequisite for making consultations inclusive or fun was to create a positive association with coming to the clinic.

‘I really want them to leave here, thinking that it was ok to be in the hospital. Because, of course I know, that sometimes they must have blood samples done as well. Luckily, it’s not here with me, it’s over at the lab, right. So it’s not always fun to come to the hospital, but it must be ok when they come into our [consultation] room, here with us. It needs to be a good place to be’. – Paediatrician (19).

The HCPs found that the education material they had available was largely not suitable for young children. Although the material included pictures and illustrations, it failed to capture young children’s attention in a way that was meaningful to them.

‘For preschool children, it can be difficult to show these pictures of the apple going down into the stomach and the pancreas situated there. [… ] So we try, as early as possible, to involve them in, in a very, very simple way, that there is some relationship between eating and what one’s blood sugar levels are’. – Nurse (1).

According to the HCPs, young children could still be actively included in education and treatment using other approaches. One HCP pointed out that all children had a wish and need to be involved in what was happening to them. The only question was how to do this appropriately.

‘They do really have a need to be involved. But involved where we have - that is, where it has been well reflected on; what are they to be involved in? And at what level? And at what point? [… ] Because they mirror what they see, and they snap up everything. That nonverbal communication, they pick it up straight away, right. So it’s really important, that when we plan our teaching and plan new initiatives, that we then also consider: How are we going to present this to the child? Yes, in my experience at least, I often find that the results are best if we’ve considered; how do we get the child involved? And in what way?’ – Nurse (13).

Many HCPs reported that fear in children was often associated with a lack of understanding of what was going to happen to them. A number of HCPs found it useful to demonstrate medical procedures on teddy bears, or even on themselves, prior to the procedure being done on the child. This practice resonated with young children, broadening their understanding and, thus, minimising fear.

‘I also often use the needle on myself, it sounds so dramatic. But if I have to do something to these children, where they’re having a sensor put on, or a pump or something, then I demonstrate it on myself. So that they can see what my face looks like when I put something into my body, that they’re about to have’. – Nurse (3).

While this approach did not always result in children happily complying with medical treatment, the HCPs found it helpful in that young children would engage in this type of interaction. Additionally, medical role-play was helpful when reconnecting with a child following an unpleasant or painful procedure. This also helped the child ‘play out’, or work through, the difficult experience as a means to move past the event.

‘And then I had to be cut on my leg with the, -because there was this knife in that doctor’s set, right? Then I had to have a bandage on. So I kind of had to go through-, I had to go through the same thing as him, right? And then we played a little bit, so that now it was his turn to kind of be the one to do things to me, right? And then it was ok. Then he was-, then he got over it quickly’. – Nurse (3).

The HCPs explained that getting young children to actively participate in the preparation of a medical procedure was a tangible way of including them in their own treatment as well as increasing their comprehension and feeling of agency. It was a way to make diabetes education meaningful and to encourage age-appropriate participation in their own care.

‘Well, it’s about, that you have to go down to the child’s level and explain the things that need to be explained, right. And why we do these things. And again, we need to have the parents on board too, because, so that the child feels more secure about the things that need to be done. We try to explain what needs to be done, nice and calmly. We’re trying to get them involved in preparing the blood glucose meter for example, put the strip in’. – Nurse (15).

While all clinics had a psychologist on the diabetes team, only a few worked directly with young children. Most would see the caregivers instead. However, psychologists who included young children in their consultation found that they added valuable insights and that children benefitted from ‘having a say’ in their own treatment.

According to the psychologists, given the right framework and tools, even young children could relate their feelings and express preferences for the support offered by their caregivers in difficult situations.

‘Yes, we draw a lot and maybe use the board, they also draw a bit on the board. We have some toys with us, but it’s not something I systematically include in the conversation, it’s a bit dependent on what it is. We have some Duplo with different emotions, so it may well be that we use them to express whether one felt sad or happy or whatever it was, in that age group’. – Psychologist (4).

‘There are also a lot of children who, for example, don’t like needles, so you work with that. We then work with drawings, and how do we get a handle on the fact that you have to be pricked or measured. So a lot is with drawings. We can also do it with play, with dolls and dollhouses, it depends on what age the child is [… ] Yes, and how do we help the child cope with pricks or injections next time. How to draw it, and who is in control? The fact that the child himself gets the experience that it is me who can accomplish this. [… ] so you know, participation within the boundaries is quite important –even for a very young child’. – Psychologist (7).

### Playful Communication

The HCPs used a combination of play, humour and positive feedback when interacting with young children in a way that was meaningful to them. Accordingly, our fourth theme describes how the HCPs used play-based approaches across all themes, whether the aim was to meet the immediate emotional needs of young children or to encourage meaningful participation. Some HCPs would get on the floor and play with cars, others would sit on children’s furniture or join young children in the playroom or designated play area. The common thread here was that they sought to meet young children in their own domain, by stepping into their world. Hence, the data revealed that the HCPs used play-based approaches to connect and interact with young children, within the ZPD, thereby making activities meaningful and relevant.

Additionally, the HCPs found that young children were very responsive to positive feedback, mainly in the form of praise, but tangible rewards such as small toys were also greatly appreciated by young children.

‘Many of them have this scanner, which they scan to get a tissue sugar and then we can ask them; how do you do it? And, can I see? and what do you do and –so we get something hands on, and they also get the opportunity to show us all the things they can do. In my opinion, that works really well and it’s completely undramatic and not scary. It can only be positive, because they can only receive praise. You can’t do anything wrong’. – Paediatrician (19).

Small toys were used as an incentive to comply with medical procedures, thus avoiding any use of force; they were also used to ensure that young children associated the clinic with something positive. The HCPs used positive feedback to reward and encourage engagement and participation. However, it was never contingent on an outcome.

‘They learn so fast, even when they’re quite young, in my experience, whether a blood sugar is good or bad, that is. And where I say, after all, they shouldn’t call it good or bad. Every blood sugar measured, I usually say to them, is a good blood sugar! But there are some we need to do something about, there are some that are higher, and we need to do something about them’. – Nurse (12).

The HCPs used a combination of play and humour to distract children before or during potentially painful procedures. They found that distraction, in combination with appropriate pain relief, could decrease fear and anxiety and defuse tension, for both the young children and their caregiver.

‘Diversions can be helpful [… ] I have this funny pen that can, there’s a kind of “TV” on, you can press a button and then Mickey appears. Then we can talk a little bit about it and maybe get a little preoccupied with it, right, and then they barely notice it. Because that “magic patch” actually works really well. Because often it’s not the pain, it’s the fear that they don’t know if it will hurt, and the thing about; if they have to be restrained. What is happening? They can also sense that mum is getting nervous and dad is nervous, right, and that they want this over and done with’. – Nurse (16).

The HCPs described how creating a playful and fun atmosphere was of great importance to young children’s experiences, as it counteracted fear and anxiety and could alter the perception of pain associated with treatment. For this purpose, the HCPs found that the assistance of hospital clowns (Hospital Clowns (Danske Hospitalsklovne) is a privately founded charity that supports hospitalised children and their families) was very helpful.

‘That you can get some things through, some procedure through without the cost being too high for the kids, regarding pain and suffering, in my opinion at least. [… ] I have some procedures, some things that are painful but that can be accomplished, with the help of [hospital] clowns. And I think that’s good. [… ] Yes, or get them laughing or something. Getting them in a good mood and stuff like that, I think’. – Paediatrician (18).

## Discussion

Analysing our data in a developmental perspective using the theory of ZPD proved valuable in understanding the care needs of young children diagnosed with type 1 diabetes. It also provided insights into why CCC matters even for young children with type 1 diabetes, and what this care may look like in practice.

The HCPs provided CCC, largely through play-based approaches and starting from the children’s tangible experiences and understandings, in this way providing the necessary scaffolding that is essential to young children’s ability to engage, comprehend and participate in their own care. Our findings are consistent with research indicating that even young children’s levels of understanding, knowledge, and skill gained from their experience of living with diabetes develop through experience rather than as a function of age (19).

The usefulness of ZPD in the area of nursing interventions for children has previously been proposed (20) and may be particularly pertinent when caring for young children. We found that operating within the ZPD, using appropriate scaffolding and playful communication were at the core of being responsive to the needs and preferences of young children, establishing therapeutic relationships, eliciting the child’s perspectives and encouraging participation.

As young children’s views and competencies in relation to diabetes care are largely missing from the literature (4, 13, 19), it is difficult to compare our result with findings from other studies. However, our findings suggest that young children, like older children and adults, have a need for and benefit greatly from CCC practices.

While the present study illustrates that HCPs can successfully accommodate young children’s preference for relationship-based care and enable young children to actively participate in their care, other research suggests that children (4-10 years of age) with type 1 diabetes do not experience the clinic environment as one in which they can usefully contribute (21). This indicates that current practices may not adequately demonstrate a belief in the value and validity of children’s views or support children’s active participation (21). Likewise, our results highlighted a disparity between clinics where HCPs were responsive to young children’s needs and preferences and clinics that waited for children to grow older before actively considering their need to participate.

This inconsistency in practices may reflect a lack of research or, more likely, a failure to consider the child’s perspective in the research and, thus, in guidelines and recommendations. While the present study represents a child perspective, it does not include the *child’s* perspective, which embodies the child’s insider perspective on experiences, perceptions and actions, based on what the child deems important (10). Both perspectives are necessary if we are to better understand the value of CCC and should be given due consideration in guidelines and recommendations. Indeed, if we are to live up to the UN Convention on the Rights of the Child, at a minimum, children should be listened to, supported in expressing their views and have their views taken into account (22).

Research indicates that HCPs find it difficult to meet children’s preferences for collaborative, relationship-based care (13). Therefore, identifying and describing existing, tangible practices that have been successfully integrated into clinics may be particularly useful for diabetes teams and HCPs working with young children. Our results showed that current educational material was primarily aimed at older children. Besides age-appropriate educational tools, tools that can aid communication between young children and HCPs are needed. Developing such tools in cooperation with young children and HCPs could be a useful strategy for encouraging and facilitating CCC in this age group. We suggest that future research include the perspective of young children, directly and through observation. We argue that including the voices of young children who have been diagnosed with type 1 diabetes will add valuable information about their experiences and needs.

### Implications for Practice

Our findings point to specific CCC practices that can be incorporated into diabetes care for young children. Such practices will encourage care that is responsive to the needs and preferences of young children, foster relationships, and frame diabetes within the context of children’s everyday lives.

- Use play-based approaches and positive feedback to build relationships, minimize anxiety, elicit the child’s perspective and encourage participation.
- Designate a time when young children can contribute to the consultation, or if hesitant, gently encourage them to practise this skill, using playful communication and age-appropriate tools. Clearly define activities in which children can actively contribute and participate, as well as activities aimed at their caregivers.
- View diabetes within the context of children’s everyday lives, by making notes about the things children chose to share about their lives, allowing you and others to do follow-ups.
- Prioritize building a relationship, in order to identify preferences for support and learn how individual children best express their emotions.

### Strengths and Limitations

The main limitation of the present study is that our results are not based on actual observation of current practices. We recognize that there may be a discrepancy between described practices and actual practices. Likewise, self-selection of HCPs, with an interest in psychosocial aspect of diabetes care, is a potential source of selection bias. Therefore, our result may not accurately represent current diabetes practice. Further, this study solely explored the child perspective though the HCPs position. Young children and their parent’s perspective on successful CCC practices are not represented in this study. A key strength of the present article is the broad inclusion and geographical representation of all regions in Denmark. Through our approach of identifying existing practices, we ensure that our results are relevant and can be integrated into current clinical practice.

## Data Availability Statement

The datasets presented in this article are not readily available because consent for sharing interview data has not been obtained from participants. Requests to access the datasets should be directed to pd@psy.ku.dk.

## Ethics Statement

The studies involving human participants were reviewed and approved by The Institutional Ethical Review Board, University of Copenhagen, Department of Psychology. The patients/participants provided their written informed consent to participate in this study.

## Author Contributions

PD, DG, and TS contributed to the conception and design of the study, PD collected and transcribed the data and drafted the manuscript. PD led the analysis and interpretation of data in collaboration with DG, LJ, and TS. DG, LJ and TS critically revised the article for intellectual content and gave their approval of the final version to be published.

## Conflict of Interest

The authors declare that the research was conducted in the absence of any commercial or financial relationships that could be construed as a potential conflict of interest.

## Publisher’s Note

All claims expressed in this article are solely those of the authors and do not necessarily represent those of their affiliated organizations, or those of the publisher, the editors and the reviewers. Any product that may be evaluated in this article, or claim that may be made by its manufacturer, is not guaranteed or endorsed by the publisher.
